# Supplementary material for: Loss of Rnf31 and Vps4b sensitizes pancreatic cancer to T cell-mediated killing
Source: Nat Commun. 2022 Apr 4;13:1804. doi: 10.1038/s41467-022-29412-3 (PMC8980030; doi:10.1038/s41467-022-29412-3)
Supplement: Supplementary file 1 — Supplementary Information [file 41467_2022_29412_MOESM1_ESM.pdf]

Supplementary information

"Loss of Rnf31 and Vps4b sensitizes pancreatic cancer to T cell-mediated killing "

Nina Frey[1,2], Luigi Tortola[1], David Egli[1], Sharan Janjuha[2], Tanja Rothgangl[2], Kim Fabiano Marquart[1,2], Franziska Ampenberger[1], Manfred Kopf[1] and Gerald Schwank[1,2]*

*Corresponding author: gerald.schwank@uzh.ch

Affiliations:

1 Institute of Molecular Health Sciences, ETH Zurich, Zurich, Switzerland

2 Department of Pharmacology and Toxicology, University of Zurich, Zurich, Switzerland

This PDF file includes:

Supplementary Figures 1 – 6

Supplementary Tables 1 – 3

Supplementary References

Supplementary Figures

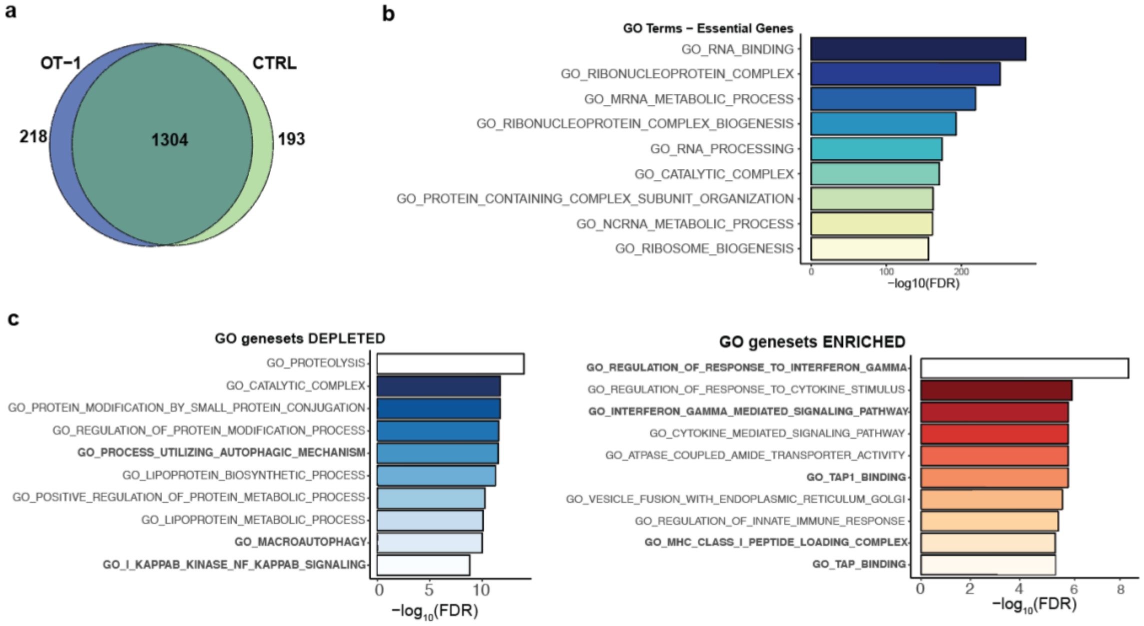

Supplementary Figure 1. Related to Figure 1: Genome-wide CRISPR screen unravels immune evasion mechanisms in PDA. (a) Venn diagram of essential genes in KPC-OVA cells untreated (CTRL) or after 3d OT-1 T cell treatment (OT-I). Guide RNA abundance was compared to plasmid Brie library using MAGeCK to find essential genes. **(b)** Pathway analysis of significantly depleted genes in CTRL and OT-I conditions (1304 genes, see S1A) using the Molecular Signature Database (MSigDB). **(c)** Pathway analysis of significantly enriched/depleted genes in OT-I treated vs. untreated KPC cells (FDR < 0.1) using the Molecular Signature Database (MSigDB). The -log10 FDR of the top ten pathways are represented. Source data are provided as a Source Data file.

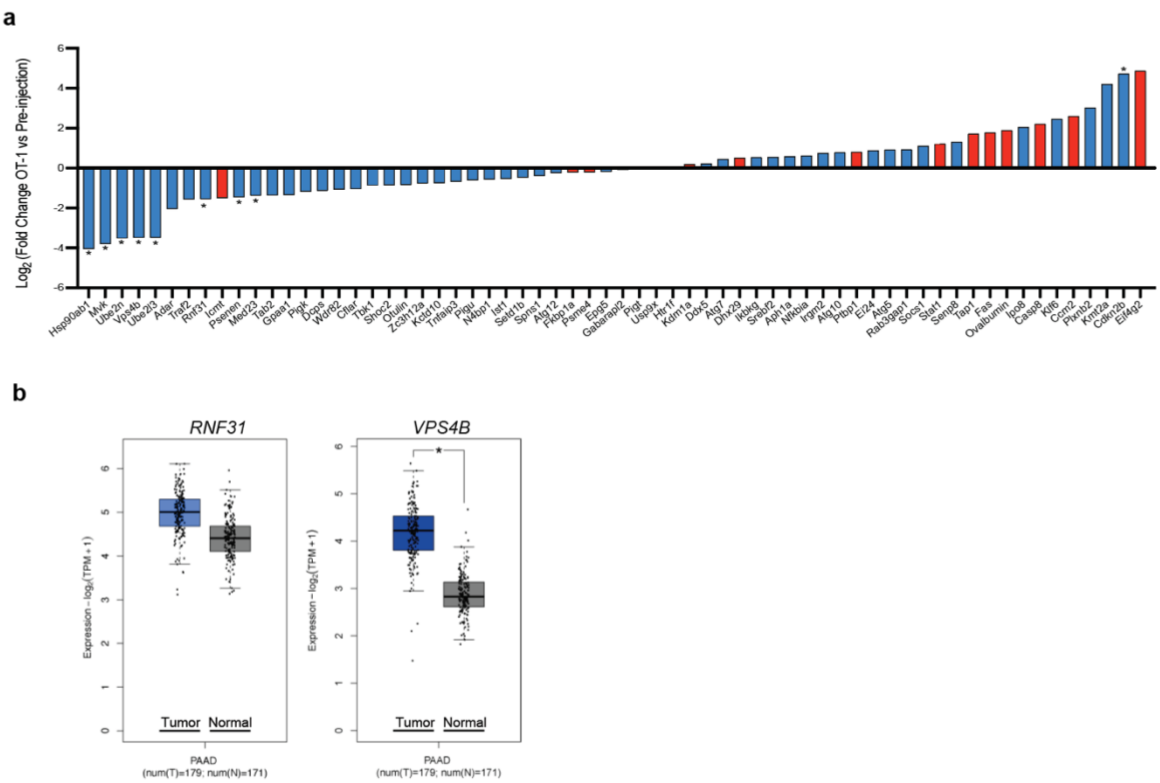

Supplementary Figure 2: Related to Figure 2: In vivo CRISPR screening validates hits identified in the in vitro CRISPR screen. (a) Log_2Fold change of all sublibrary genes (OT-I treated mice vs. pre-injection pool of cells using MAGeCK). Red and blue indicates predicted to be enriched or depleted based on genome-wide in vitro screen, respectively. Asterisks indicate FDR < 0.2. (**b**) Human expression data of *RNF31* and *VPS4B* in pancreatic adenocarcinoma (PAAD). Tumor data was retrieved from TCGA, normal tissue data from TCGA and GTEx. Plot and analysis were done using the platform GEPIA2[3]. For statistical analysis the p value cut off was set to < 0.01 for significance. The middle line in the boxplots shows the median, the lower and upper hinges represent the first and third quartiles, and whiskers represent ±1.5× the interquartile range. Source data are provided as a Source Data file.

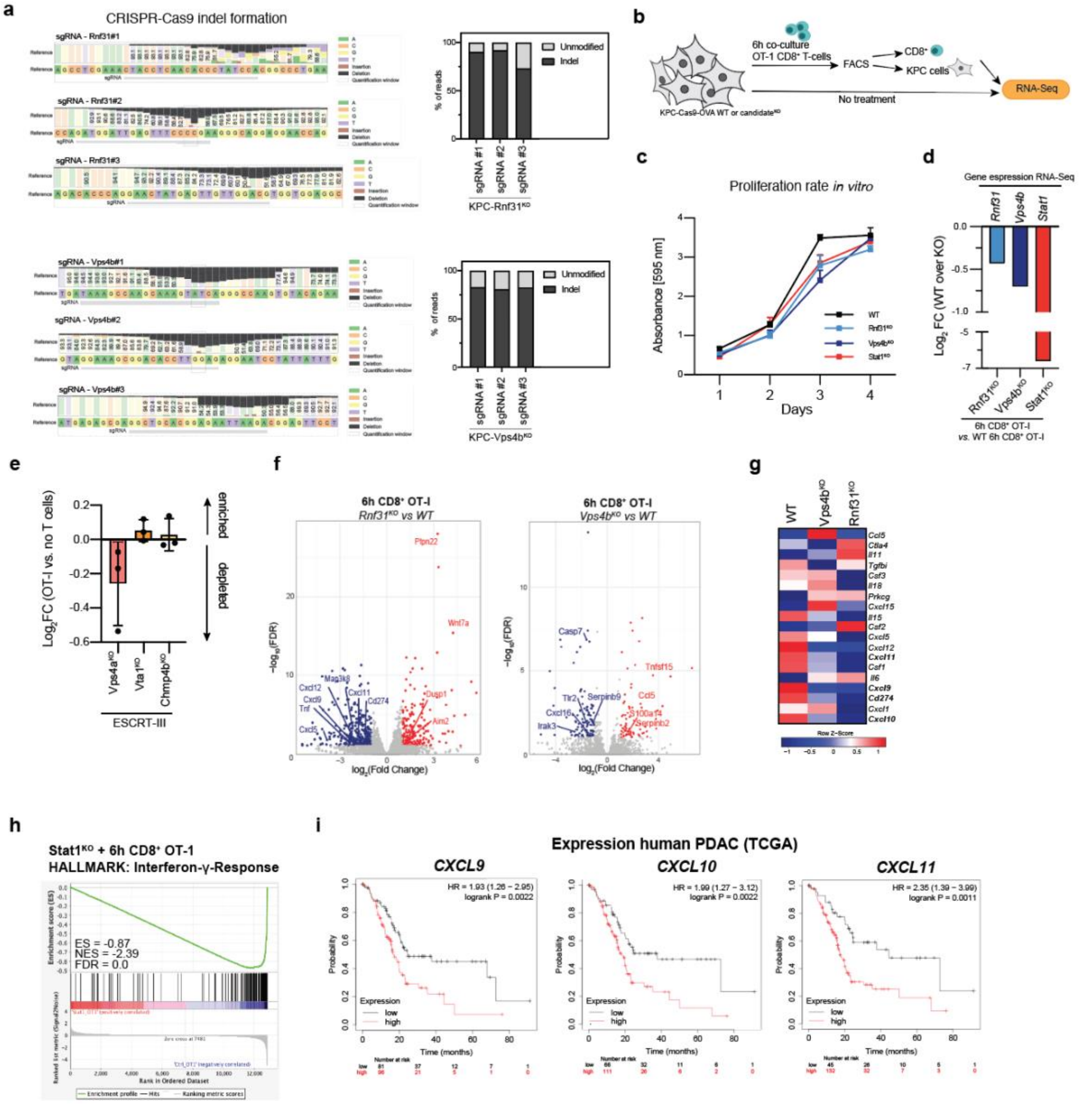

Supplementary Figure 3. Related to Figure 3: *Rnf31*^{KO} and *Vps4b*^{KO} alter transcriptional response upon T cell exposure. (a) Targeted amplicon sequencing of cut site of each sgRNA targeting *Rnf31* or *Vps4b*. Left panel shows percentage of individual base modifications, panel of the right shows overall percentage of indel frequency at the respective target site per sgRNA. **(b)** Schematic of workflow for RNA Sequencing sample generation. **(c)** Proliferation rate of different engineered KPC-1 cell lines. Crystal violet staining was carried out and normalized to day 1 from 6 technical replicates. Values represent mean ± SD. **(d)** Log$_2$ fold change of candidate genes expression compared to KPC-1 control cells. **(e)** Log$_2$ Fold change of mCherry$^+$ KPC population before and after OT-I co-culture with sgRNAs targeting different components of the ESCRT-III complex. Values represent mean ± SD, n = 3 independent experiments. **(f)** Volcano plots of differentially expressed (DE) genes in *Rnf31*^{KO} and *Vps4b*^{KO} cells after 6h of OT-I T cell exposure compared to equivalently treated KPC^{WT} cells.

Highlighted genes are putatively involved in anti-tumor immunity. DE genes in red/blue: | Log_2FC | > 1, FDR < 0.1. **(g)** Heatmap of normalized counts per million (CPM) of selected immune modulatory factors after OT-I T cell exposure across different genotypes. (**h**) Gene set enrichment analysis (GSEA) of RNA-Seq data in $Stat1^{KO}$ KPC cells after 6h OT-I co-culture compared to WT _ OT-I. **(i)** Kaplan-Meier plot of human pancreatic cancer cases (n = 177) from the cancer genome atlas (TCGA) analyzed according to the platform "Kaplan-Meier Plotter" [1]. Source data are provided as a Source Data file.

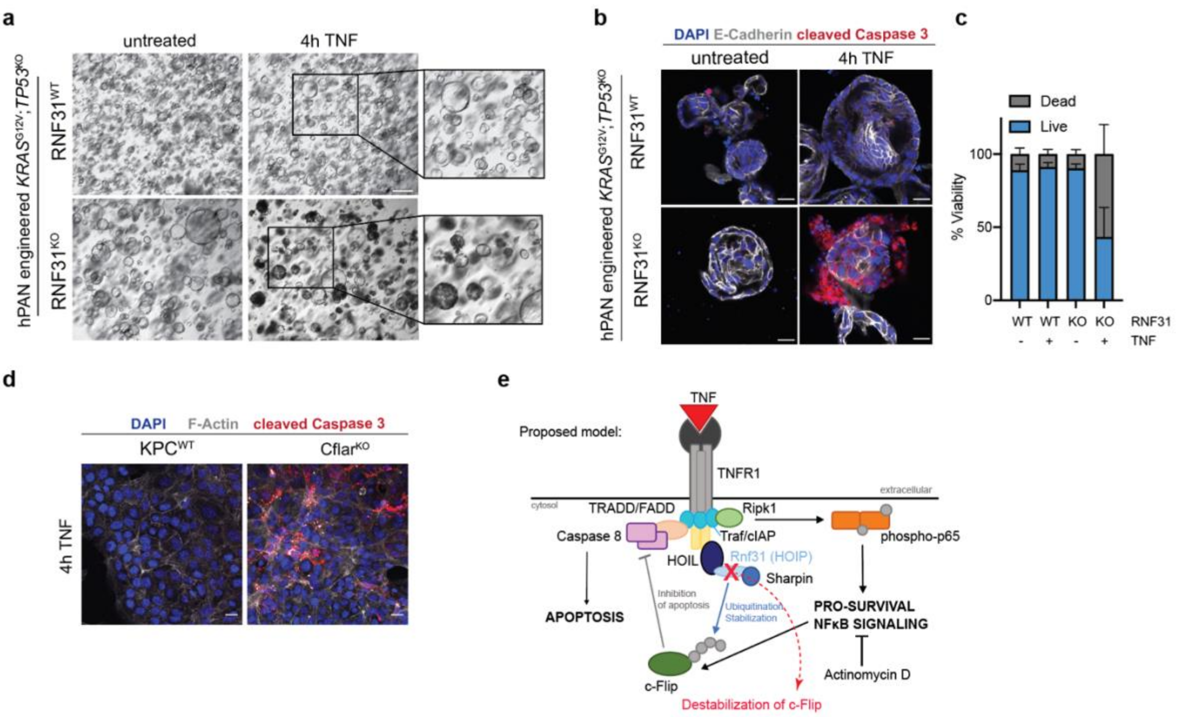

Supplementary Figure 4. Related to Figure 4: *Rnf31*^{KO} sensitizes PDA to TNF-induced apoptosis in human 3D organoids. (a) Brightfield images of human engineered pancreatic organoids in the presence of 100 ng/ml TNF for 4h. Boxes highlight viable and dying organoids. Scale bar represents 200 µm. **(b)** Whole mount staining of engineered human pancreatic organoids after 4h TNF (100 ng/ml) treatment with cleaved caspase 3 (red), E-cadherin (white) and DAPI (blue). Scale bar represents 20 µm. **(c)** Quantification of **(b)** Relative organoid viability, individual organoids were counted and classified in 'live' or 'dead'. Percentage dead/live of the counted area of all organoids is displayed. Values represent mean ± SD, n = 3 independent experiments. **(d)** Immunofluorescence staining of KPC-WT and *Cflar*^{KO} (coding for c-Flip) after 4h of 100 ng/ml TNF. Cleaved caspase 3 (red), F-Actin (grey) and DAPI (blue). Scale bar represents 20 µm. Immunofluorescence stainings and bright field images in (**a**), (**b**) and (**d**) were repeated three times on independent samples. Representative images are shown. **(e)** Schematic of TNF-induced signaling cascade with emphasis on LUBAC. Adapted from Tang *et al.* 2018 [2]. Source data are provided as a Source Data file.

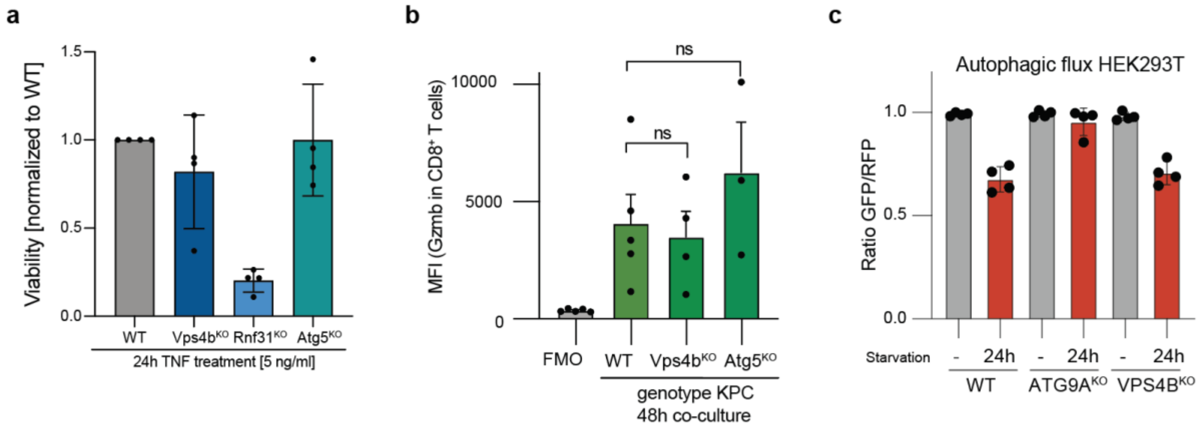

Supplementary Figure 5. Related to Figure 5: *Vps4b^{KO}* abrogates autophagy and increases granzyme B levels. **(a)** Relative viability (normalized to KPC-WT cells) after 24h treatment with 5 ng/ml TNF without Actinomycin D. Values represent mean ± SD, data are derived from four independent experiments. **(b)** Mean fluorescence intensity (MFI) of granzyme B contents in CD8^{+} OT-I T cells. FMO (Fluorescence Minus One) control for Gzmb-FITC antibody. Significance was determined with one-way ANOVA; ns, non-significant, $p > 0.05$. Values represent mean ± SEM, data are derived from n=5 (FMO, WT), n=4 (Vps4b) and n=3 (Atg5) independent experiments. **(c)** Quantification of autophagic flux by flow cytometry in HEK293T cells under normal and starvation conditions (24h in EBSS + 2% FBS). Bars represent the ratio of GFP to RFP expressing cells. Values represent mean ± SD, data are derived from four independent experiments. Source data are provided as a Source Data file.

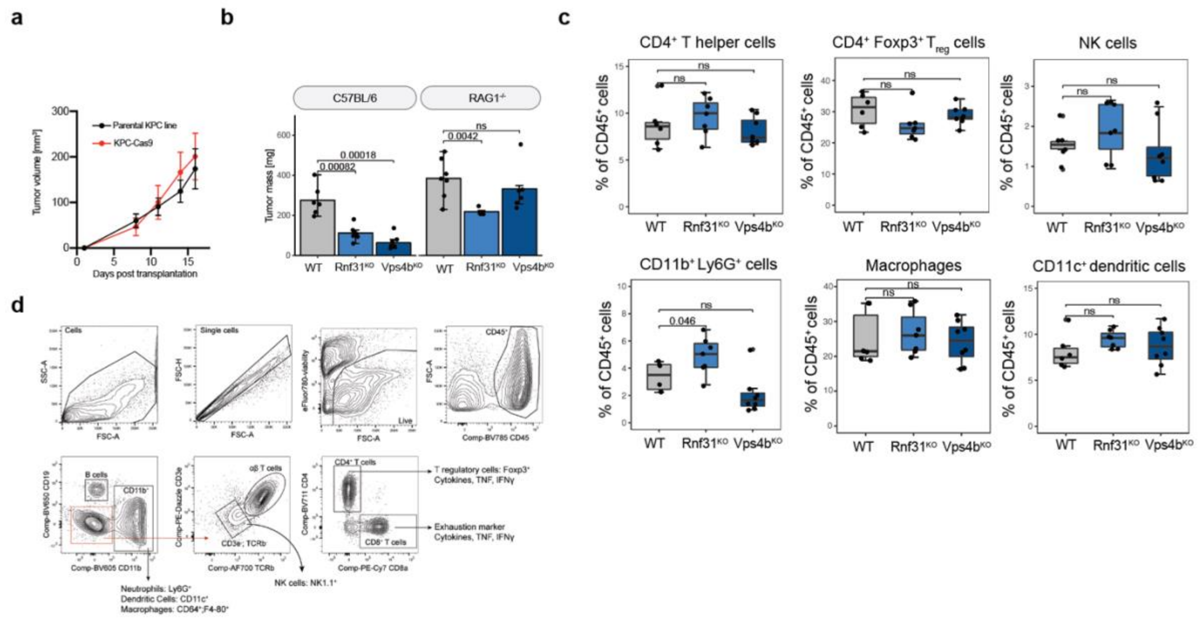

Supplementary Figure 6. Related to Figure 6: *Rnf31*^{KO} and *Vps4b*^{KO} alter tumor growth *in vivo*. (a) Tumor growth of subcutaneous KPC (black) and KPC-Cas9 (red) tumors into C57BL/6 mice; bi-flank injection in five mice per group. Values represent mean ± SD**. (b)** Tumor mass of candidate KPC line in immune competent C57BL/6 mice and RAG1$^{-/-}$ mice. C57BL/6: n = 6 (WT); 7 (*Rnf31*^{KO}); 8 (*Vps4b*^{KO}); RAG1$^{-/-}$: n = 7 (WT), 5 (*Rnf31*^{KO}); 6 (*Vps4b*^{KO}). Values represent mean ± SD**.** Significance was determined with an unpaired, two-tailed t-test. ns, non-significant, $p > 0.05$. **(c)** Flow cytometry analysis of immune cell population within tumors (n for WT = 6; *Rnf31*^{KO} = 7; *Vps4b*^{KO} = 8). Significance was determined with an unpaired two-tailed t test. ns, non-significant, $p > 0.05$. The middle line in the boxplots shows the median, the lower and upper hinges represent the first and third quartiles, and whiskers represent ±1.5× the interquartile range. **(d)** Gating strategy for TME characterization. Source data are provided as a Source Data file.

Supplementary Table 1: Gene summary (MAGeCK RRA) of top candidate genes in genome-wide OT-I screen (FDR < 0.1). OT-I treated vs untreated KPC cells.

		Positive Selection		Negative Selection	
**Gene**	**LogFC**	**p value**	**FDR**	**p value**	**FDR**
Ifngr2	2.2254	2.40E-07	0.00055	1	1
Ifngr1	2.3361	2.40E-07	0.00055	1	1
Stat1	2.2376	2.40E-07	0.00055	1	1
Jak1	2.204	2.40E-07	0.00055	1	1
Jak2	1.6873	2.40E-07	0.00055	1	1
Ccm2	0.93447	2.40E-07	0.00055	1	1
Kdm1a	0.95777	2.40E-07	0.00055	1	1
Casp8	1.0021	2.40E-07	0.00055	1	1
Fkbp1a	1.0791	2.40E-07	0.00055	1	1
Ptbp1	0.83122	4.07E-06	0.007651	0.99986	1
Tap1	0.83631	4.07E-06	0.007651	0.99999	1
Icmt	0.69433	5.51E-06	0.009488	1	1
Eif4g2	0.62934	2.08E-05	0.032885	0.99986	1
Tapbp	0.71959	2.23E-05	0.032885	0.99999	1
Tap2	0.65265	3.23E-05	0.044554	0.99999	1
Fas	0.69197	3.66E-05	0.047339	0.99913	1
Psme4	0.7215	6.78E-05	0.082411	0.99993	1
Rnf31	-1.3877	1	1	2.40E-07	0.000381
Vps4b	-1.2716	1	1	2.40E-07	0.000381
Gabarapl2	-0.86886	1	1	2.40E-07	0.000381
Klf6	-0.62437	1	1	2.40E-07	0.000381
Tnfaip3	-0.95731	1	1	2.40E-07	0.000381
Gpaa1	-1.0891	1	1	2.40E-07	0.000381
Ube2n	-1.1611	1	1	2.40E-07	0.000381
Cflar	-0.91552	1	1	2.40E-07	0.000381
Atg5	-1.115	0.99997	1	2.40E-07	0.000381
Aph1a	-0.85653	0.99634	1	2.40E-07	0.000381
Adar	-1.1541	0.99829	1	2.40E-07	0.000381
Ei24	-1.1046	0.99973	1	2.40E-07	0.000381
Srebf2	-0.93756	0.99691	1	2.40E-07	0.000381
Irgm2	-0.92123	1	1	7.19E-07	0.00099
Shoc2	-0.83244	1	1	7.19E-07	0.00099
Tab2	-1.0876	0.99973	1	1.20E-06	0.001456
Traf2	-0.8403	0.99996	1	1.20E-06	0.001456
Nfkbia	-0.60428	1	1	2.87E-06	0.0033
Usp9x	-0.71683	1	1	4.07E-06	0.004429
Pigk	-0.58995	1	1	4.55E-06	0.004479
Ube2l3	-0.95439	1	1	4.55E-06	0.004479
Kctd10	-0.65842	1	1	5.51E-06	0.005176
Tbk1	-0.82417	0.90318	1	5.99E-06	0.005381
Cdkn2b	-0.43096	1	1	6.95E-06	0.005982
Med23	-0.79003	1	1	7.43E-06	0.006139
Epg5	-0.69529	0.79229	1	8.38E-06	0.006664
Atg12	-0.9694	0.95429	1	9.34E-06	0.007151
Ddx5	-0.87276	1	1	9.82E-06	0.007249
Socs1	-0.96168	0.83603	1	1.27E-05	0.009047
Atg10	-0.69371	1	1	1.65E-05	0.011386
Ist1	-0.79476	0.99131	1	1.94E-05	0.012935
N4bp1	-0.5666	0.99999	1	3.09E-05	0.019652
Pigt	-0.76161	0.99992	1	3.14E-05	0.019652
Setd1b	-0.61491	0.99999	1	3.23E-05	0.019656
Spns1	-0.4862	0.99998	1	5.01E-05	0.029562

Kmt2a	-0.53789	0.99998	1	5.58E-05	0.032041
Atg7	-0.67195	0.22399	0.998633	6.30E-05	0.035189
Zc3h12a	-0.53973	0.99974	1	8.74E-05	0.047551
Hsp90ab1	-0.94902	0.90062	1	9.41E-05	0.049886
Plxnb2	0.33279	0.017553	0.811154	9.99E-05	0.051609
Ipo8	-0.47609	0.92711	1	0.00012048	0.060734
Ikbkg	-0.62624	0.85092	1	0.00012719	0.062514
Pigu	-0.66735	0.99982	1	0.00013006	0.062514
Psenen	-0.6673	0.99203	1	0.00015833	0.07437
Rab3gap1	-0.44779	0.8693	1	0.00016455	0.075578
Mvk	-0.77305	0.99815	1	0.00018419	0.082759
Senp8	-0.54521	0.99176	1	0.00019473	0.085633
Htr1f	-0.44009	0.99994	1	0.00021964	0.094575
Otulin	-0.6721	0.99994	1	0.00023018	0.09709

Supplementary Table 2: List of genes in the targeted CRISPR sublibrary based on top candidates of the genome-wide *in vitro* screen (FDR < 0.1)

Gene	Number of sgRNAs	Prediction GWS screen
Casp8	10	Enriched
Ccm2	10	Enriched
Eif4g2	10	Enriched
Fas	10	Enriched
Fkbp1a	10	Enriched
Icmt	10	Enriched
Kdm1a	10	Enriched
Ovalbumin	7	Enriched
Psme4	10	Enriched
Ptbp1	10	Enriched
Stat1	10	Enriched
Tap1	10	Enriched
Adar	10	Depleted
Aph1a	10	Depleted
Atg10	10	Depleted
Atg12	10	Depleted
Atg5	10	Depleted
Atg7	10	Depleted
Cdkn2b	10	Depleted
Cflar	10	Depleted
Dcps	10	Depleted
Ddx5	10	Depleted
Dhx29	10	Depleted
Ei24	10	Depleted
Epg5	10	Depleted
Gabarapl2	10	Depleted
Gpaa1	10	Depleted
Hsp90ab1	10	Depleted
Htr1f	10	Depleted
Ikbkg	10	Depleted
Ipo8	10	Depleted
Irgm2	10	Depleted
Ist1	10	Depleted
Kctd10	10	Depleted
Klf6	10	Depleted
Kmt2a	10	Depleted
Med23	10	Depleted
Mvk	10	Depleted
N4bp1	10	Depleted
Nfkbia	10	Depleted
Otulin	10	Depleted
Pigk	10	Depleted
Pigt	10	Depleted
Pigu	10	Depleted
Plxnb2	10	Depleted
Psenen	10	Depleted
Rab3gap1	10	Depleted
Rnf31	10	Depleted
Senp8	10	Depleted
Setd1b	10	Depleted
Shoc2	10	Depleted
Socs1	10	Depleted
Spns1	10	Depleted

Srebf2	10	Depleted
Tab2	10	Depleted
Tbk1	10	Depleted
Tnfaip3	10	Depleted
Traf2	10	Depleted
Ube2l3	10	Depleted
Ube2n	10	Depleted
Usp9x	10	Depleted
Vps4b	10	Depleted
Wdr82*	10	Depleted
Zc3h12a	10	Depleted
Non-targeting	600	Depleted
**Total:**	**1237**	

Supplementary Table 3: Gene summary (MAGeCK RRA) of sublibrary in vivo screen. OT-I treated mice vs. plasmid library.

		Positive Selection		Negative Selection	
**Gene**	**LogFC**	**p value**	**FDR**	**p value**	**FDR**
Vps4b	-3.4933	0.57416	1	4.95E-06	0.001096
Hsp90ab1	-4.0424	0.96674	1	4.95E-06	0.001096
Ube2l3	-3.4897	0.89437	1	4.95E-06	0.001096
Mvk	-3.7923	0.99984	1	1.49E-05	0.001974
Ube2n	-3.5023	0.35716	1	1.49E-05	0.001974
Rnf31	-1.5502	0.9635	1	0.00019321	0.021382
Psenen	-1.4586	0.98627	1	0.00023284	0.022086
Dcps	-1.1319	0.99124	1	0.0021847	0.161915
Med23	-1.3727	0.44142	1	0.0021946	0.161915
Adar	-2.042	0.9033	1	0.0044339	0.294408
Tab2	-1.3684	0.57417	1	0.023636	1
Traf2	-1.5665	0.46052	1	0.024498	1
Icmt	-1.4927	0.24827	1	0.028411	1
Pigk	-1.1718	0.73845	1	0.028441	1
Cflar	-1.0461	0.55064	1	0.031998	1
Gpaa1	-1.3499	0.71803	1	0.039181	1
Shoc2	-0.85451	0.26075	1	0.05763	1
Wdr82	-1.0617	0.99689	1	0.067558	1
Tbk1	-0.87305	0.98235	1	0.072998	1
Pigu	-0.60473	0.8428	1	0.07708	1
Tnfaip3	-0.67185	0.87792	1	0.11683	1
Otulin	-0.84912	0.28649	1	0.14763	1
Kctd10	-0.74863	0.35715	1	0.1867	1
Epg5	-0.1858	0.28649	1	0.19712	1
N4bp1	-0.56002	0.50731	1	0.20553	1
Setd1b	-0.47321	0.28649	1	0.21116	1
Zc3h12a	-0.76704	0.86264	1	0.30423	1
Ist1	-0.53971	0.35716	1	0.30496	1
Irgm2	0.75237	0.27347	1	0.33906	1
Pigt	-0.038195	0.80842	1	0.34589	1
Atg7	0.45524	0.2865	1	0.35474	1
Nfkbia	0.62486	0.55065	1	0.35567	1
Psme4	-0.20224	0.31057	1	0.3936	1
Spns1	-0.3847	0.64684	1	0.40478	1
Usp9x	0.0098218	0.68419	1	0.42424	1
Ikbkg	0.54739	0.64685	1	0.44576	1
Atg12	-0.24401	0.46052	1	0.54292	1
Kdm1a	0.19833	0.75799	1	0.55631	1
Fkbp1a	-0.21733	0.44141	1	0.56106	1
Srebf2	0.55871	0.12455	1	0.61809	1
Gabarapl2	-0.085073	0.80445	1	0.6301	1
Rab3gap1	0.93038	0.46051	1	0.64833	1
Htr1f	0.072868	0.98078	1	0.64922	1
Ddx5	0.22954	0.35716	1	0.80202	1
Atg10	0.79672	0.73324	1	0.81679	1
Dhx29	0.51731	0.87303	1	0.85282	1
Ei24	0.88513	0.016294	1	0.88165	1
Klf6	2.4688	0.26076	1	0.88862	1
Socs1	1.1162	0.45111	1	0.9505	1
Ovalbumin	1.8922	0.35239	1	0.94762	1
Tap1	1.7225	0.2865	1	0.9632	1
Plxnb2	3.0225	0.18298	1	0.98228	1

Aph1a	0.58394	0.71802	1	0.98492	1
Atg5	0.9242	0.56681	1	0.99067	1
Casp8	2.216	0.27347	1	0.99158	1
Eif4g2	4.8799	0.13904	1	0.9936	1
Senp8	1.3182	0.35715	1	0.99397	1
Ptbp1	0.81344	0.56681	1	0.99769	1
Stat1	1.22	0.24826	1	0.99962	1
Fas	1.7888	0.26076	1	0.99972	1
Ipo8	2.0565	0.18299	1	0.99973	1
Ccm2	2.6031	0.2986	1	0.99997	1
Cdkn2b	4.7317	0.00013376	0.088816	1	1
Kmt2a	4.2092	0.047801	1	1	1

Supplementary References

1. Nagy, Á., Munkácsy, G. & Győrffy, B. Pancancer survival analysis of cancer hallmark genes. *Sci. Rep.* **11**, 6047 (2021).
2. Tang, Y. *et al.* Linear ubiquitination of cFLIP induced by LUBAC contributes to TNF-induced apoptosis. *J. Biol. Chem.* **293**, 20062–20072 (2018).
3. Tang, Z., Kang, B., Li, C., Chen, T. & Zhang, Z. GEPIA2: an enhanced web server for large-scale expression profiling and interactive analysis. *Nucleic Acids Res.* **47**, W556 (2019).
